# Clinical Presentation of Agenesis of Corpus Callosum Among Children in a Single Tertiary Care Center in Riyadh, Saudi Arabia

**DOI:** 10.7759/cureus.102041

**Published:** 2026-01-21

**Authors:** Waleed Altwaijri, Reem S Bajaman, Manal G Almutairi, Asma A Alomary, Sarah K Al Zuhair, Layan O Alhudaif

**Affiliations:** 1 Pediatric Neurology, King Abdullah Specialized Children’s Hospital, Riyadh, SAU; 2 Medicine and Surgery, King Saud Bin Abdulaziz University for Health Sciences College of Medicine, Riyadh, SAU; 3 Medicine, King Saud Bin Abdulaziz University for Health Sciences College of Medicine, Riyadh, SAU

**Keywords:** agenesis of corpus callosum (acc), comorbidities, corpus callosum, corpus callosum in children, partial

## Abstract

One of the most prevalent brain structural disorders is agenesis of the corpus callosum (ACC). ACC happens when the primary commissural fiber tracts that link the brain hemispheres are absent entirely or partially due to a disturbance of neural cell migration during fetal growth. This research was conducted based on an analytical case-series design. Specifically, a chart review was done by using the patients' charts in King Abdullah Specialized Children's Hospital (KASCH), a tertiary governmental hospital in Riyadh, Saudi Arabia, from 2015 to 2022. A non-random, non-probability consecutive sampling technique was used for this research. Thirty-seven patients were included, comprising 16 males (43.2%) and 21 females (56.8%). The ages ranged from two to 13, with 14 patients being below five years old (37.8%), 20 patients between five years old and 10 years old (54.1%), and three patients above 10 years old (8.1%). Dysmorphic features were present in 25 patients (67.6%), developmental delay in 21 patients (56.8%), seizures in 10 patients (27%), vision impairment in 12 patients (32.4%), and hearing impairment in four patients (10.8%). Cardiac comorbidities were found in 18 patients (48.6%), gastrointestinal comorbidities in 18 patients (48.6%), respiratory comorbidities in 15 patients (40.5%), neurological comorbidities in 26 patients (70.3%), and nephrological comorbidities in 10 patients (27%). MRI was used as a diagnostic tool in 28 patients (75.7%), while nine patients (24.3%) used other diagnostic methods. All comorbidities do not have any significant association with the type of ACC. However, rarely does ACC occur in an isolated manner.

## Introduction

One of the most prevalent brain structural disorders is agenesis of the corpus callosum (ACC) [1]. Its incidence has been estimated to range from 1.8 per 10,000 live births to 230-600 per 10,000 in children, and it is linked to neurodevelopmental impairment [2]. During pregnancy, the corpus callosum develops from front to back, starting at eight weeks and continuing until 20 weeks. After birth, the corpus callosum continues to mature [3]. ACC happens when the primary commissural fiber tracts that link the brain hemispheres are absent entirely or partially due to a disturbance of neural cell migration during fetal growth [4,5]. The source of ACC is unknown [3]. However, it may be caused by many factors, including prenatal infections, maternal drinking, and genetic mutations. Moreover, there are many genes that were linked to ACC [6]. For example, a rare autosomal recessive disorder that was associated with ACC is Andermann syndrome [7].

Most cases of ACC are asymptomatic [8]. However, the most common signs and symptoms in individuals might include seizures, hearing or vision loss, headaches, issues with hand-eye coordination, and hydrocephalus [9]. ACC shows a decreased interhemispheric transfer of sensory motor information, decreased speed of cognitive processing, and difficulty with complicated processing [10]. There are several types of ACC, and they can be classified through two different methods. The first method can be categorized based on the pathophysiology [9]. Type one happens when the axons form but are incapable of crossing the midline. As a result, they form large abnormal Probst bundle fibers parallel to the medial hemispheric walls [9]. Type two occurs when the commissural axons are unable to form, so Probst fiber bundles are not found. Another way to categorize ACC is based on the final effect on the corpus callosum: complete agenesis or partial ACC. There is no treatment for ACC; however, the symptoms presented can be managed palliatively [9]. 

ACC is usually detected during routine prenatal checkups; if there is a suspicion of a brain defect, then additional investigations would be ordered. They include an anatomy ultrasound and a fetal MRI, which is considered the gold standard [11]. The anatomy ultrasound would be used to confirm the presence of an anomaly and to look for other anomalies, and an MRI is used to provide further information on the baby’s overall development [12].

According to Hussen et al.’s study, which was conducted in the national research center in Cairo, Egypt, from 2016 to 2019, the study implemented a cohort-based study of all patients that presented with various phenotypes associated with corpus callosum abnormalities in order to determine the prevalence and characteristics of chromosomal abnormalities among these patients, in addition to the subsequent imperative diagnostic steps to reach a precise diagnosis to provide proper management and genetic counseling [13]. The sample size was 105 patients with no exclusion criteria. Further classification divides the sample into 56 men and 49 women [13]. Furthermore, a detailed history taking with clinical examination, psychological assessments, neuroimaging studies, and cytogenetic studies. The results showed that developmental delays were indicated in the majority of patients (74%), followed by intellectual disability (50.4%), dysmorphic features (34%), and microcephaly (28%) [7]. In addition, based on MRI results, patients were classified into four categories: agenesis, hypoplasia, dysplasia, and dysplasia with hypoplasia together. This research is considered weak in representing the clinical presentation, as it doesn’t shed light on other brain abnormalities that could affect the signs and symptoms that a patient could present with. Furthermore, the clinical presentation might differ in Saudi Arabia on the basis of demographics [13].

Previous studies have also described the clinical and diagnostic profiles of ACC, highlighting its variable presentations and outcomes across different populations [14]. Moreover, radiological and clinical correlations have been emphasized in recent literature, showing how imaging findings can predict neurological development and assist in patient management [15].

In conclusion, most of the literature about ACC comes from the West. There is very limited published literature on ACC from Saudi Arabia. Therefore, this research aims to identify the clinical presentation of patients with isolated ACC and with other brain abnormalities among children in the tertiary hospital, King Abdullah Specialized Children’s Hospital (KASCH), Riyadh, from 2015 to 2022.

## Materials and methods

This research was conducted based on an analytical case-series design. Specifically, a chart review was conducted using the patients' charts in KASCH, a tertiary governmental hospital, from 2015 to 2022. Thirty-seven patients were included. The inclusion criteria are the following: male and female patients and neonates until the age of 14 years old, as well as all patients diagnosed with ACC, were included. The main outcome variables that are identified include signs and symptoms, type of agenesis, symptomatic management options, and comorbidities. The secondary variables include gender and age. A non-random, non-probability consecutive sampling technique was used for this research. Data entry was entered into Microsoft Excel (Microsoft Corp., Redmond, WA, USA), and the statistical analysis for the data was conducted using the statistical program IBM SPSS Statistics software version 26.0 (IBM Corp., Armonk, NY, USA). Numerical variables, such as age, were depicted as the mean. While the rest of the categorical data, like gender, comorbidities, etc., were depicted as percentages and frequencies. The chi-square (X²) test was used to assess the relationship between the main outcome variables and baseline characteristics of patients.

Complete ACC refers to the total absence of the corpus callosum, with no interhemispheric commissural fibers visualized on imaging. Partial agenesis refers to incomplete formation of the corpus callosum or hypoplasia, where some callosal fibers are present but do not fully connect both hemispheres.

## Results

Thirty-seven patients were included in the study, all of Saudi Arabian descent, 16 male (43.2%) and 21 female (56.8%), with 14 patients being below five years old (37.8%), 20 patients between five and 10 (54.1%), and three patients above 10 years old (8.1%). The mean age of the sample was 5.51 years old. Twenty-two patients had a complete ACC (59.5%), while 15 patients had partial ACC (40.5%).

**Figure 1:**
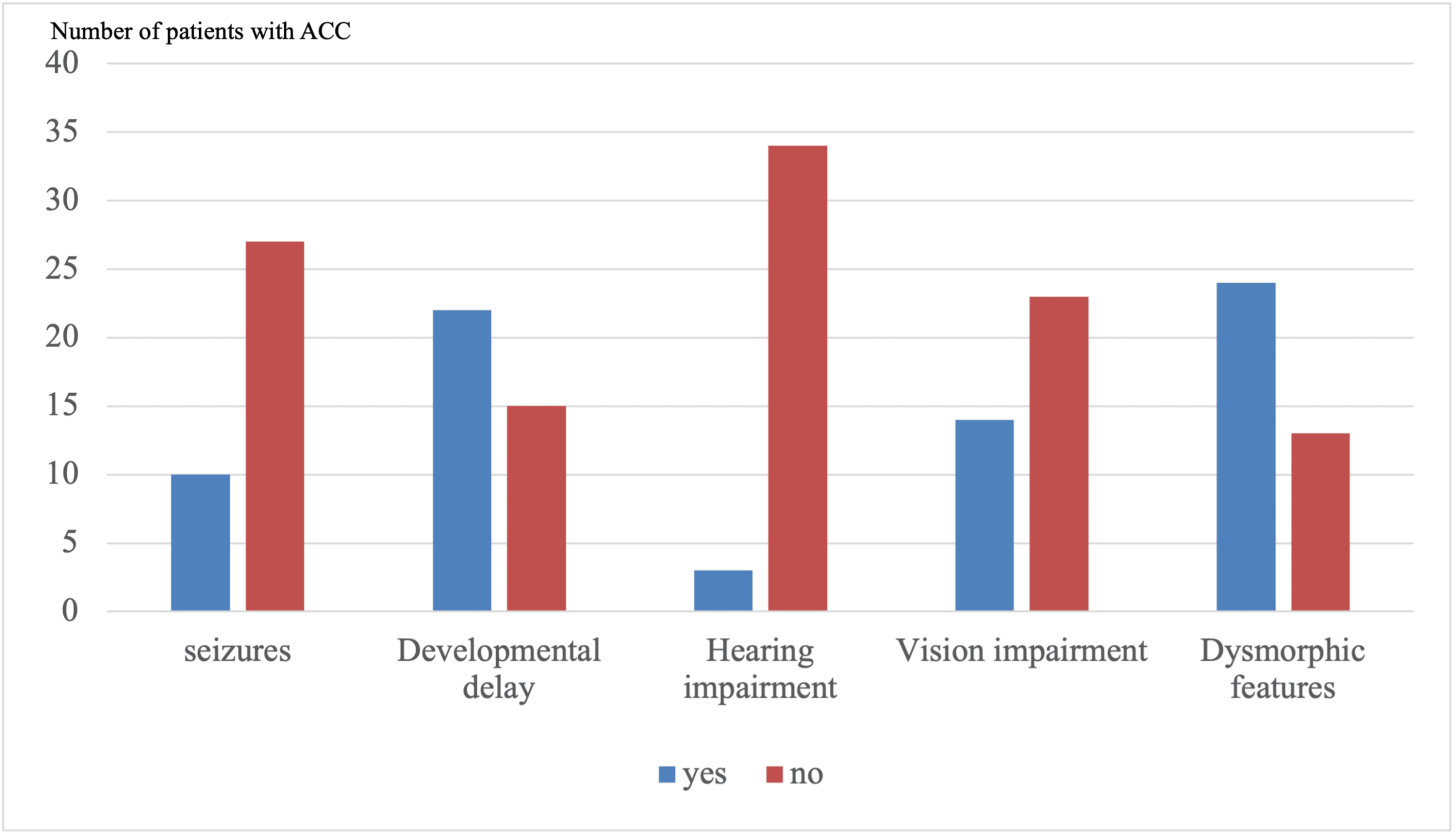
Signs and symptoms of patients affected with agenesis of the corpus callosum (ACC)

Observed clinical features included dysmorphic features in 25 patients (67.6%), with microcephaly being the most common feature; developmental delay in 21 patients (56.8%); seizures in 10 patients (27%); vision impairment in 12 patients (32.4%), mainly strabismus; and hearing impairment in four patients (10.8%) (Figure 1). Other concurrent comorbidities that were found included cardiac comorbidities in 18 patients (48.6%), with congenital heart disease present in seven of the 18 patients; gastrointestinal comorbidities in 18 patients (48.6%), with gastroesophageal reflux disease present in eight patients; respiratory comorbidities in 15 patients (40.5%), with asthma being the most occurring illness in seven patients; neurological comorbidities in 26 patients (70.3%), with hydrocephalus being the most common in seven patients; and 10 (27%) out of 37 patients had nephrological comorbidities, and five out of 10 patients had hydronephrosis. 

MRI was used as a diagnostic tool in 28 patients (75.7%), while nine patients (24.3%) used other diagnostic methods such as CT scans or sonography. On average, patients started getting diagnosed at 12 months, with the youngest patient being three days old and the oldest patient being eight years old. Five patients got diagnosed after completing their first year of life (13.5%), 19 patients got diagnosed before completing their first year of life (51.4%), and 13 patients got diagnosed before completing the first month of life.

All medications prescribed were used to treat symptoms and preexisting conditions patients suffered from. The most commonly prescribed was acetaminophen for pain relief in 23 patients (62.2%); second was chloral hydrate as a sedative in 14 patients (37.8%); and lastly, cholecalciferol for vitamin D deficiency in nine patients (24.3%).

X² analysis was used to identify the presence of a relation between comorbidities and ACC. Observed cardiac comorbidities (X²= 0.222, P= 0.638), gastrointestinal comorbidities (X²= 0.040, P= 0.842), respiratory comorbidities (X²= 1.713, P= 0.191), neurological comorbidities (X²= 0.157, P= 0.692), and nephrological comorbidities (X²= 0.002, P= 0.967). Therefore, all comorbidities have shown no association with the disease. The analysis showed no significant association between the disease and cardiac, gastrointestinal, respiratory, neurological, and nephrological associations (Table 1).

**Table 1:** The association of different comorbidities and the type of corpus callosum agenesis (complete, 22 patients; partial, 15 patients), as well as the chi-square values.

Type of comorbidities	Complete agenesis of the corpus callosum (22 patients)	Partial agenesis of the corpus callosum (15 patients)	Chi-square value	P-value
Cardiac comorbidities	10	8	0.222	0.638
Gastrointestinal comorbidities	11	7	0.04	0.842
Respiratory comorbidities	7	8	1.713	0.191
Neurological comorbidities	16	10	0.157	0.692
Nephrological comorbidities	6	4	0.002	0.967

## Discussion

This study aims to assess the clinical features of isolated ACC and other brain conditions among 37 patients up to the age of 14 in KASCH, a tertiary hospital in Riyadh, from the year 2015 to 2022. Patients were differentiated into two groups: complete ACC representing the majority, in comparison to partial agenesis of the corpus callosum. Moreover, thinning and hypoplasia of the corpus callosum were considered as partial agenesis. This classification was done based on diagnostic modalities, including MRI, the main diagnostic tool, and CT and sonography if MRI was not applicable. 

Furthermore, out of 37 patients, strikingly, female incidence was at a higher rate, which is opposed to Hussen et al. and Shevell’s samples, in which there was a higher male incidence at 53.3% and 62.5%, respectively. The reason for this variation is thought to be due to genetic or environmental factors [13, 14]. However, similar to Hussen et al. and Shevell, in this study, the pattern of presenting symptoms showed similar numbers, including first dysmorphic features in the majority of the patients, with microcephaly being the most common feature, followed by developmental delay, then seizures, and finally vision impairment presenting mainly with strabismus and hearing impairment [13,14]. In addition, Hussen et al. show that developmental delays are indicated in the majority of patients (74%), followed by dysmorphic features (34%), and finally seizures (10.4%) [13]. Shevel's sample displayed the majority of patients having developmental delay at 83.3%, then dysmorphic features at 50% [14].

Regarding the associated abnormalities, neurological, including hydrocephalus, was the most common; cardiac and gastrointestinal were equally in second place; respiratory was in third; and nephrological was the least common, with the musculoskeletal comorbidities being evaluated. In terms of the management options, supportive therapy was initiated to control other comorbidities. Similarly, Al‐Hashim et al.’s study involved system involvements other than the brain in subjects with the following percentages: gastrointestinal (37%), genitourinary (34%), heart (21%), lung (13%), and musculoskeletal (34%) [15].

This research has presented new points of view, from the wide range of variables and the differing geographical locations, as it highlights the scarcity of existing literature regarding ACC in Saudi Arabia. The conduction of this study as a case series further strengthens the results, as it emphasizes the importance of diagnosing the rare disorders based on the clinical manifestation, which has a huge role in improving the management and care provided by the clinician. Furthermore, the analysis of the data by using the IBM SPSS platform, which is one of the most precise, reliable, and valid analysis programs, raises and supports the quality of the provided results. 

The generalizability of this research is limited due to the small size of the sample, as it is conducted in a single setting. The lack of genetic testing in the source (Bestcare) and the correlation between it and ACC could lead to a different and new perspective.

## Conclusions

To sum up, this research contributes to the understanding of ACC in the Saudi Arabian population, highlighting the clinical heterogeneity, diagnostic criteria, and management strategies associated with ACC. It could be complete ACC or partial, associated with other comorbidities, or isolated. It can present with no symptoms, which may not affect the patient’s daily lifestyle, or it can present with a multitude of concomitant abnormalities. The relationship between ACC and associated comorbidities remains variable, and further research is required to better understand potential associations. As a result, there is no relationship between the ACC and other comorbidities. Nevertheless, further studies with larger sample sizes and longitudinal follow-up are warranted to validate these findings and explore the genetic and environmental factors contributing to ACC in Saudi Arabia. In addition, it will lead the clinicians in the management plan and increase the level of awareness and knowledge about ACC.
